# Advancements in Research on Prevention and Control Strategies for Maize White Spot Disease

**DOI:** 10.3390/genes14112061

**Published:** 2023-11-10

**Authors:** Enyun Xing, Xingming Fan, Fuyan Jiang, Yudong Zhang

**Affiliations:** 1 Institute of Resource Plants, Yunnan University, Kunming 650500, China; enyun18266462838@163.com; 2 Institute of Food Crops, Yunnan Academy of Agricultural Sciences, Kunming 650205, China; jiangfuyansxx@126.com (F.J.); mikezhangy@yahoo.com (Y.Z.)

**Keywords:** maize white spot disease, *Pantoea ananatis*, gene mapping, transmission pathway, prevention and control strategies

## Abstract

Maize white spot (MWS), caused by the bacterium *Pantoea ananatis*, is a serious disease that significantly impacts maize production and productivity. In recent years, outbreaks of white spot disease have resulted in substantial maize yield losses in southwest China. Researchers from various countries worldwide have conducted extensive research on this pathogen, including its isolation and identification, the localization of resistance genes, transmission pathways, as well as potential control measures. However, the information related to this disease remains fragmented, and standardized preventive and control strategies have not yet been established. In light of this, this review aims to comprehensively summarize the research findings on MWS, providing valuable insights into understanding its occurrence, prevention, and control measures in the southwestern and southern regions of China while also mitigating the detrimental impact and losses caused by MWS on maize production in China and across the world.

## 1. Introduction

Maize is an important crop with widespread cultivation worldwide, ranking third in terms of cultivated area, following wheat and rice. Maize serves as a vital source of food, feed, and raw materials for various industries. Particularly in economically disadvantaged areas, maize plays a significant role not only in providing food security and clothing but also in contributing to employment and the generation of income in impoverished regions. Unfortunately, in recent years, maize production has continuously suffered huge losses due to the damage caused by maize white spot disease (MWS). In China, MWS outbreaks are frequent during maize growing seasons, and without timely intervention, they can severely impact production, especially in the southern regions of the country.

MWS has a global presence and is prevalent in Central and South America, South Asia, and Africa, primarily in tropical and subtropical regions, with limited reports from other areas. Previous studies on MWS have yielded varying results concerning the root causes of the pathogen responsible for MWS. Some studies have identified MWS-resistant gene loci in different maize germplasm resources at home and abroad, while other studies suggest that the disease’s transmission routes may be complex and diverse. To enhance our understanding of the MWS-causing pathogen, effective disease control measures, and the current research status on genetic control mechanisms, this review aims to compile and summarize existing MWS-related research findings. This review could assist farmers in implementing effective strategies to mitigate the damage caused by MWS and lay a foundation for further scientific research on MWS in the southwest and southern regions of China or across the world.

### Occurrence, Influencing Factors and Severity of MWS in Maize 

The occurrence of MWS was first documented in India [1]. Subsequently it emerged in Brazil during the 1980s, spreading rapidly across nearly all maize-producing regions in the country [2]. Over the following years, MWS rapidly disseminated and induced disease outbreaks in agricultural regions across Central and South American countries. In the 1990s, leaf spot was initially identified in winter breeding nurseries located in South Florida, USA [3]. 

Initially, leaf spot disease in Brazil was referred to as Phaeosphaeria leaf spot (PLS). However, extensive research conducted by Paccola-Meirelles et al. (2001) from the United States and other countries led to the identification of the pathogen responsible for this disease [2]. Consequently, this disease was reclassified as MWS (hereafter, MWS is used). During the early 2000s, there was a substantial surge in the prevalence of MWS across several African countries, including Zimbabwe, Kenya, and Cameroon. Notably, South Africa, as the largest maize producer in the region, experienced severe epidemics [4,5,6]. Furthermore, MWS has also been reported in other regions such as Mexico, Argentina, Poland, Europe, and Ecuador [7,8,9,10]. Starting in July 2020, MWS was first detected in scattered locations in southwest and southern China, rapidly spreading to multiple provinces in south China. Surprisingly, within a few weeks, a substantial portion of the maize crop became infected, resulting in rapid and severe damage to the crop in numerous provinces, with major maize varieties being particularly affected. Since then, the disease has persistently spread from the middle and high-elevation regions of the tropics to surrounding areas in recent years, exhibiting a further expansion trend in China [11]. In southwest China, the average incidence rates of plants with diseases ranged from 42.7% to 100%, while the average leaf infection rate ranged from 39.12% to 88.6%, resulting in yield losses of 10% to 50% in corn production. Additionally, MWS has significantly impacted certain varieties of sweetcorn [12,13], resulting in a substantial decrease in income for farmers.

Historical data indicate that the emergence of new leaf spot infections tends to have detrimental effects on crop yield [14]. Several reports have demonstrated that severe leaf spot infections could result in a yield reduction of over 60% in maize production in Brazil and a 13% decline in maize yield in the USA [5,15]. Furthermore, it is worth noting that for every one percent increase in disease severity, the yield and grain weight of susceptible maize hybrids decreases by 0.23% and 0.16%, respectively [16]. The reasons for these yield losses in maize infected by MWS could be attributed to several factors. Firstly, MWS infection causes the wilting and whitening of leaf tissue, leading to a reduction in the photosynthetic area and weakened nitrogen transport capacity in maize. In particular, before the maize filling period, if more than one-tenth of leaves are infected, it can result in a substantial 40% decrease in the net photosynthetic rate. Consequently, premature leaf senescence occurs, and the reproductive maturity stage ends prematurely, thereby shortening the entire life cycle of maize [17,18]. Additionally, leaf damage hinders the sufficient accumulation of organic matter in plants and limits the effective expression of grain length and weight potential [19,20], ultimately resulting in a decline in corn yield. Furthermore, maize is typically infected by the MWS pathogen both before and after flowering, with peak incidence occurring during the grain-filling stage or milk-ripening stage [6]. This results in a notable disparity in the chemical composition between infected and healthy leaves, disrupting normal plant tissues and physiological processes related to chemical element metabolism [5,21]. Consequently, it becomes challenging for affected maize plants to achieve their normal production level.

Capucho et al. (2010) developed and validated a diagrammatic scale method to measure MWS infection [22]. They established the disease severity scale using leaves with both the maximum and minimum disease severity, calculating the median value. Subsequently, they photographed leaves with varying disease severity, grouped them based on the same disease grade, and employed a software tool to calculate the disease area. They constructed a linear model using expected disease scales and true severity scales to evaluate the accuracy and precision of the disease severity by analyzing error variance [22]. The author referred to it as a composite linear model based on image matching. This model has greatly enabled researchers to conduct epidemiological research on MWS with greater accuracy. In China, the MWS severity assessment in maize is primarily conducted via a visual evaluation of experienced researchers. The severity of maize loss in a region is usually influenced by factors such as the extent of maize cultivation and the intensity of MWS. Numerous studies have shown that the primary internal factors affecting severity are the inherent resistance of maize genotypes to this disease and their defense mechanisms against microorganisms and pests [23,24]. 

Primary external factors include general climatic conditions, particularly temperature and humidity fluctuations during the maize growth cycle [25]. Prolonged extreme weather events in a region can induce variations in environmental temperature and humidity, thereby impacting the microbial ecology, including bacteria, fungi, and viruses as well as the physiological responses of maize plants at different developmental stages. These changes may also alter host susceptibility or their interactions with pathogens, resulting in shifts in survival strategies, reproductive patterns, and nutritional modes across various organisms [24]. Under new ecological conditions, the simultaneous occurrence of multiple diseases or even the emergence of novel disease complexes can be facilitated more easily. Additionally, the duration of surface humidity and relative humidity constitutes a crucial environmental factor in the pathogenesis and progression of numerous plant pathogens [26]. This is why MWS is commonly observed in complex environments characterized by consistent rainfall and moderate temperatures.

## 2. Pathogen and Characteristics of MWS

### 2.1. Pathogens

Since the emergence of MWS, it has posed significant threats to maize cultivation in tropical and subtropical regions. Continuous studies worldwide have aimed to isolate and identify the disease pathogens. However, a consensus on this causative pathogen has not yet been reached. Initially, it was believed that the disease was caused by *Phaeosphaeria maydis*, a necrotic fungus-causing corn leaf spots, which was easily isolated from disease spots in Brazil, the United States, and other locations. While these studies are widely accepted, they are also limited in their number of pathogen re-inoculation and successful tests conducted [4,16,19]. Cervelatti et al. (2002) compared the conidia of fungi with ascomycetous shells isolated from the diseased spot and found that the sources of the two were distinct, with putrefactive fungi also present in the center of the lesion [27].

Another perspective suggests that *Pantoea ananas*, a bacterial pathogen of maize, is the causative agent of MWS [2,28,29]. During the cytological analysis of bacteria associated with MWS, a colony-producing yellow pigment was isolated from early lesions and identified as *Pantoea ananas* (synonymous with *Erwinia ananas*), which is implicated in the initial stage [2,23]. An abundance of bacteria was observed under electron microscopy in the initial phases of both artificial and natural infection lesions, with no fungal structures or dark leaf ball cavity bacteria present. Fungal hyphae are only seen in tissues that undergo necrosis during intermediate and advanced stages, and up to a dozen fungal species might be isolated [21]. Further studies confirmed the presence of *Pantoea ananas* in nearly all stages of MWS development [29]. Lanza et al. (2009) isolated and tested the pathogenicity of *Pantoea ananatis* and *Phaeospeeria maydis*, the two prime suspected pathogens of MWS, in a greenhouse [30]. Plants inoculated with *P. ananatis* exhibited typical MWS symptoms. Subsequently, *Pantoea ananatis* was re-isolated from these lesions. The affected spots on collected leaves underwent sequencing and molecular validation, revealing a gene sequence with 99% similarity to *Pantoea ananatis*. This evidence confirmed that *Pantoea ananatis* caused the observed disease. Subsequent studies, based on Koch’s postulates, yielded consistent results [27,28]. However, multiple attempts to validate Koch’s hypothesis using *P. maydis* were unsuccessful [30,31]. The consensus among the aforementioned researchers is that *Pantoea ananatis* is the primary pathogenic bacteria, with fungi easily isolated from plaque but not the main cause of MWS.

A different group of scholars observed that early MWS infections displayed infiltrative changes rather than necrotic spots. However, the bacteria remained dormant, causing tissue destruction through proliferation. Over time, the bacterial population decreased while the number of fungi increased. Fungal species began to colonize the pre-existing lesions caused by *Pantoea ananatis* [16,31]. This explains why fungi are easily isolated from these lesions. Amaral et al. (2005) conducted a secondary disinfection treatment on the samples to eliminate the effects of saprophytic bacteria [32]. The results showed that three fungi (*Phyllosticta* sp., *Phoma sorgina*, and *Spormiella* sp.) were the primary causative agents of PLS. They suggested that the geographic location and timing of corn planting could alter the composition of pathogenic bacteria in the environment, resulting in necrotic lesions caused by various microorganisms, primarily fungi. The progression of MWS was thought to be due to the combined effects of multiple pathogens [32].

*Epicoccum* sp. was recently identified as the predominant strain associated with MWS following the sampling of maize production areas in southwest and south China [33]. Researchers collected diseased tissue in Yunnan Province for isolation and purification, ultimately classifying the bacterial strain as *Epicoccum latusicolum*. The authors suggested naming the disease based on its symptoms and labelling it as MWS in their study, asserting it as a unique plant disease separate from that caused by *Pantoea ananatis* [13]. Furthermore, studies have detected *Pantoea ananatis* on the surfaces of healthy corn leaves. However, when these leaves are disinfected beforehand, the detection rate of this bacterium drops significantly, suggesting that *Pantoea ananatis* might only be an epiphyte on healthy leaves [34].

The genus *Pantoea ananatis* is characterized by its low variability, high adaptability, and potent pathogenicity across diverse environments and ecological regions [15]. The biochemical analysis of MWS bacteria initially documented in Poland revealed that four bacteria share close phylogenetic ties with many *Pantoea ananatis* strains [9]. Lana et al. (2012) performed a cluster analysis on *Pantoea ananatis* isolated from maize, demonstrating their genetic similarity ranging from 60% and 90% with *Pantoea ananatis* strains associated with sorghum MWS. After rep-PCR analysis, the eight *P*. *ananatis* isolates appeared nearly identical [35]. Furthermore, the pan-genome of *P*. *ananatis* contains numerous gene-encoding proteins that enhance its ability to colonize, survive, and affect a broad spectrum of plant and animal hosts [36]. *P*. *ananatis* is known to cause several plant diseases, including onion center rot, meshed melon internal fruit rot, sorghum leaf spot, and rice sheath rot [37,38,39,40,41]. These data furnish substantial experimental evidence to support *P*. *ananatis* as a pathogenic agent.

In conclusion, the dominant perspective among international scholars suggests that *Pantoea ananas* is the primary causative agent behind MWS. However, some contend that MWS might result from the combined effects of multiple pathogens. *Pantoea ananatis* might be one of the benign pathogens on healthy leaves, invading plant tissues only after external factors damage these leaves. Chinese experts believe that corn MWS is distinct from the ailment induced by *Pantoea ananatis*. Thus, there is no unanimous consensus on the precise pathogen responsible for MWS.

### 2.2. Features of the Lesions

**Figure 1:**
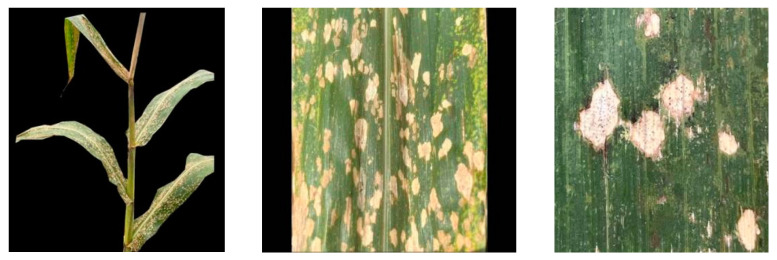
Distribution and enlargement of lesions of MWS.

Naturally infected MWS initially appears as water-soaked lesions, which then transition from dark green to white leaf spots. These spots subsequently progress into necrosis and desiccation, ultimately turning straw-colored. These lesions are primarily round or oval, though some irregular shapes are also present [23]. Most hybrids develop disease spots on the leaves below the ear, although some may appear above the ear [4]. The severity of plant infection varies. At the peak of the disease, these spots can rapidly spread across all upper-leaf surfaces, with fewer instances on the stem compared to the leaves [5,15,34]. In southwestern China, the disease first appeared in mid-July in both 2021 and 2022, reaching its peak from August to September [12,13]. In our research group’s corn experimental field in Wenshan, Yunnan Province, significant outbreaks of MWS were observed from late August to early September (Figure 1). In some susceptible groups, large-scale outbreaks occurred within a week of the disease’s appearance, particularly during the corn-filling and maturing stages, severely affecting corn cultivation. We speculate that the environmental conditions during this period were highly conducive to the reproduction of the pathogenic bacteria responsible for this disease. It is likely that one or more bacteria were involved, as fungal pathogens typically do not cause serious diseases shortly after contact with plants under normal circumstances.

## 3. Plant Immunity against Pathogens 

**Figure 2:**
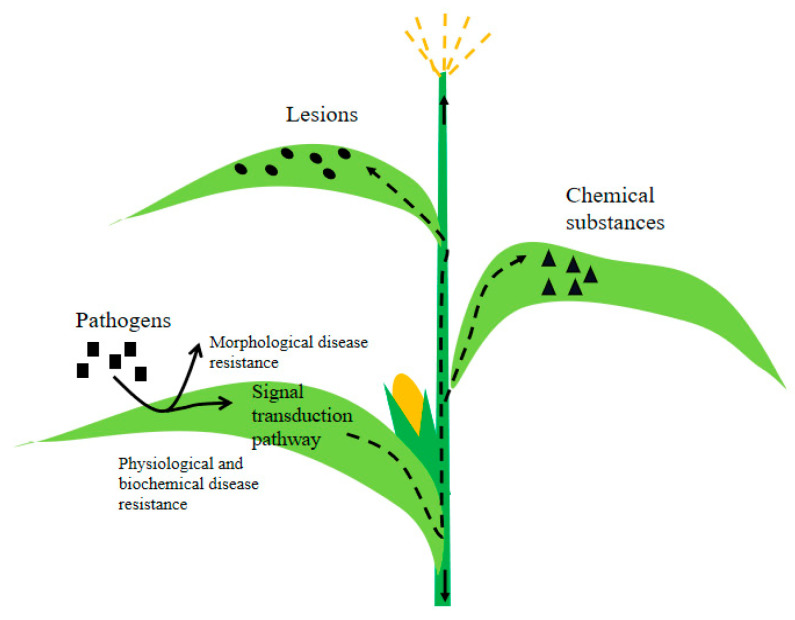
Schematic diagram of plant disease resistance mechanism.

The mechanisms of plant disease resistance fall into the following two main categories: morphological disease resistance and physiological and biochemical disease resistance (Figure 2). The former involves plants leveraging their structural components, such as the epidermis, cuticle layer, and stomatal structure, to fend off pathogens. The latter entails plants activating enzyme systems to kickstart defense mechanisms through signal transduction pathways, resulting in the production of chemical compounds or signaling molecules that inhibit the growth and spread of pathogens [42]. This process can also involve the binding of the pathogen’s *Avr* protein to the plant cell’s receptor (*R* protein), triggering localized hypersensitive reactions that destroy the plant’s tissues, forming necrotic lesions to quarantine the pathogen.

Previous studies have proposed that models categorizing the plant immune system primarily encompass two distinct branches [43], namely PTI (PAMP-Triggered Immunity) and ETI (Effector-Triggered Immunity). PTI recognizes the immune response initiated by components such as pathogen flagellin or chitin through PRRs (Pattern Recognition Receptors). Upon the inhibition of this pathogen’s process of infection, effector protein molecules are secreted by the pathogen to disrupt receptor recognition. At this stage, intracellular immune receptors, known as NLRs (nucleotide-binding leucine-rich repeat receptors), directly or indirectly recognize pathogen-specific effector proteins and resistance genes, encoding R proteins that trigger specific immune responses in plants. This process is known as ETI [44,45]. In plants, the primary PRRs are predominantly RLKs (receptor-like kinases), which represent the largest and most diverse protein superfamily in the plant kingdom and play a pivotal role in perceiving and responding to pathogenic stimuli.

For example, LysM proteins are commonly found in the structural domains of plant proteins, facilitating pathogen recognition and the initiation of immune responses. Upon oligomerization, they activate receptor-like cytoplasmic kinases and downstream MAPK (mitogen-activated protein kinase) cascade reactions to transmit signals [46]. NLRs are ubiquitously distributed across various subcellular compartments within cells, playing a pivotal role in the regulation and resistance of gene expression [47]. The PTI and ETI systems are interdependent and collectively regulate the moderate immune response in plants through a combination of positive and negative feedback mechanisms [48]. Plant resistance genes can be primarily categorized into five distinct groups as follows: NBS-LRR, protein kinases, LRR-TM, LRR-TM kinases, and inactivated toxins. Additionally, defense genes enhance the disease resistance response to various pathogens in plants, as exemplified by *EDS1*, which is a pivotal defense gene identified in Arabidopsis [49]. The specific recognition of coding products for plant disease resistance genes and non-toxic gene coding products in pathogens elicits a cascade of signal transductions, thereby triggering the onset of plant disease [50]. The *WRKY1* gene may exert a negative regulatory effect in response to the pathogen *Pst.DC3000* via the SA signaling pathway [51].

Researchers have conducted extensive studies on the mechanisms of disease resistance associated with numerous functional R genes that have been cloned. The mechanisms of plant resistance to pathogens can be broadly categorized into the following two branches: perception and loss of susceptibility, based on distinguishing events inside and outside the cell, without distinguishing between ETI and PTI. Plant resistance to pathogens is believed to be driven by nine molecular mechanisms: either the (1) direct or (2) indirect perception of pathogen-derived molecules on the cell surface by receptor-like proteins and receptor-like kinases; (3) the direct or (4) indirect intracellular detection of pathogen-derived molecules by nucleotide-binding, leucine-rich repeat receptors, or (5) detection through integrated domains; (6) the perception of transcription activator-like effectors through the activation of executor genes; and (7) active, (8) passive, or (9) host reprogramming and mediated loss of susceptibility [52].

The first R gene to be successfully cloned was *Hm1* from maize, as published in 1922. This gene encodes an enzyme that can detoxify the Helmintosporium carbonum (HC) toxin, reducing the damage caused by maize leaf blighting and ear mold [53]. Arabidopsis RLP *RBPG1*, the protein encoded by the rice R gene *Xa21*, and the tomato R gene *Ve1* can directly sense extracellular fungal polygalacturonase, such as the RaxX effector of Riceomonas and verticillium wilt effector AVE1, respectively [54,55,56]. However, the tomato R gene product Cf-2 must act through the intermediate gene *Rcr3* to recognize the fungal effector Avr2 and the nematode effector GrVap1 [57,58].

For instance, the effector ATR1 in Arabidopsis can recognize the parasitoid spore RPP1 in vivo, triggering an immune response [59]. The tobacco NLR recognition of the tobacco Mosaic virus (TMV) requires the participation of the chloroplast-localized sulfur transferase NRIP1. The pre-recognition of the transferase in complex with the effector of the helicase (p50) domain of the virus can activate the immune process [60]. Sensing AvrPhB via RPS5 requires the stimulatory effect of the exposed ring structure in the host protein PBS1 [61]. In plants, NLR can modify their recognition specificity and enhance the recognition ability of different pathogens through small changes in the LRR region and the spatial structure located at the C terminus [59,62]. Certain executioner R genes can alter the host transcription of susceptibility factors to make plants highly resistant to pathogens. Additionally, the host can actively abandon early defenses and create a process of false neglect in the pathogen to stimulate itself and produce a more intense immune response. The control region of susceptibility factors can also be recorded [52]. The cloning of a model gene, *ZmMM1*, confers resistance to various maize leaf spot diseases, including the grey leaf spot (GLS). *ZmMM1* acts as a transcription repressor and negatively regulates the transcription of specific target genes, including *ZmMM1*-target gene 3 (*ZmMT3*), which functions as a negative regulator of plant immunity and associated cell death [63]. *ZmCCoAOMT2* is a gene on chromosome 9 that encodes a caffeoyl-coa O-methyltransferase associated with the phenylpropanoid pathway and lignin production and confers quantitative resistance to GLS. Resistance results from allelic variants that cause changes in gene expression and amino acid sequence, leading to differences in the levels of lignin and other metabolites in the phenylpropanoid pathway, as well as the regulation of apoptosis [64].

## 4. Research Progress on the Identification of Resistance Germplasm and Mapping of Resistance Genes

### 4.1. Resistant Inbred Lines

MWS is a quantitative trait primarily governed by additive genetic effects [6,65,66,67,68,69]. While dominant genes play a minor role, they remain important in certain populations [67,70]. In national and international studies on MWS disease, some germplasms have shown significant resistance. Many of these germplasms not only resist various leaf spot diseases but also offer beneficial attributes, such as a high yield or strong combining ability. These germplasm resources can also be used to identify resistant gene loci and cultivate resistant varieties that can effectively minimize the adverse effects of MWS on maize production at both molecular and population levels.

**Figure 3:**
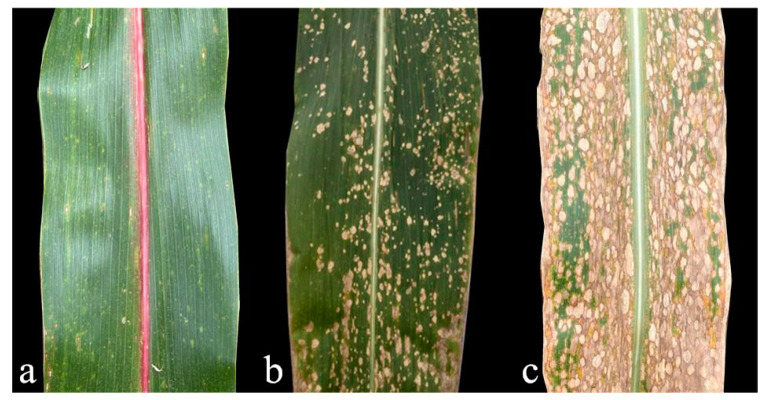
Difference in MWS resistance among different materials: (**a**–**c**) Refer to disease-resistant, susceptible, and highly susceptible maize leaves, respectively.

In Brazil, the emergence of MWS disease occurred early and became widespread, prompting an initial disease assessment and resistance identification. Investigations revealed that numerous maize lines displayed pronounced resistance to MWS, particularly specific inbred lines from the Suwan group, which showed a strong adaptability in tropical and subtropical climates. These lines also demonstrated high combining potential in terms of agronomic traits and yield. Therefore, enhancing hybrid combinations can increase their overall quality, presenting significant promise in the development of new varieties. This adaptability effectively meets the selection criteria for resistance breeding in Brazil. In the USA, American researchers evaluated the susceptibility of most corn germplasms to PLS (also known as MWS) and concluded that the natural disease was not notably threatening outside of South Florida. Even the most susceptible hybrid could experience significant grain yield reductions under artificial inoculation and conducive disease conditions [16]. However, this reduction was considerably less than in Brazil. The primary maize varieties in the United States were associated with C103 or Mo17 inbred lines, leading the author to suggest that a vast resistance resource against MWS exists within American germplasm. According to the germplasm evaluations conducted by CIMMYT and the African Center for Crop Improvement in South Africa, B23, B22, B16, CML488, and CML444 emerged as the main resistant parents of MWS-resistant hybrids. These parental lines are believed to pass on genes that provide MWS resistance to their offspring [4]. As MWS disease recently surfaced in China, corn researchers found that many domestic inbred or commercial hybrids displayed significant resistance against MWS. Zhang S et al. (2022) analyzed 622 corn samples from southwest China, determining that 65.14% of the varieties exhibited above-medium resistance levels [13]. Wang Dong and his team (2023) highlighted QR273 and QB512 as potential candidates against MWS [71]. The maize genetic and breeding group, led by Fan X M at Yunnan Academy of Agricultural Sciences in Yunnan, China, identified several inbred lines with resistance to MWS (Figure 3) that are suitable for resistance gene exploration and the development of a new variety. Currently, their group has successfully developed several commercial varieties, including Yunrui 62, Yunrui 408, Yunrui 668, and Yunrui 8, all of which demonstrate notable resistance to MWS (data not published). These varieties have been instrumental in addressing prevalent MWS in the southwest region in recent years. More details about the resistant sources are provided in the subsequent section (Table 1).

Germplasm resources showing resistance to MWS are broadly distributed worldwide, especially in Brazil, the United States, CIMMYT, the African Crop Improvement Center, and the tropical/subtropical regions of China. Utilizing these resistance materials can provide valuable insights into resistance gene mapping research and the development of new MWS-resistant varieties. PLS- or MWS-resistant inbred lines can be integrated into breeding programs, and superior commercial hybrids can be introduced and deployed in MWS-endemic regions to mitigate maize disease losses.

### 4.2. Mapping of Resistance Genes

Researchers have employed various experimental materials and methods for QTL localization, primarily utilizing techniques such as Composite Interval Mapping (CIM), Multiple Interval Mapping (MIM), and Mixed Linear Models (Table 2). Carson (2001) observed that the grade of leucoplakia in the F_2_ generation was higher than in the F_1_ generation, with the average replacement grade approximating the average grade of the backcross parents. Based on this observation, they proposed the presence of three to four resistance genes [65]. Pegoraro et al. (2002) also suggested that at least two major independent genes contribute to the inheritance of resistance for this trait [67]. In a study by Lopes et al. (2007), the tropical susceptible parent DAS21 and resistant parents DAS95 and DAS721 were used as experimental materials [66]. They identified two to three distinct genes or gene blocks and observed transgressive segregation in the F_2_ population. Additionally, they suggested that the parent DAS21, despite its susceptibility, might carry a resistance gene, facilitating the emergence of a new resistant strain from the F_2:3_ population [66,75].

While analyzing significant SNPs associated with white spot resistance in tropical maize, Rossi et al. (2020) identified the following five promising candidate gene models: GRMZM2G064580, GRMZM5G804893, GRMZM2G068331, GRMZM2G383594, and GRMZM2G10956 [77]. In a recent Chinese study, 30 QTNs related to MWS were detected across three environments in southwestern China. Out of these, 15 were common QTNs situated on chromosomes 1, 2, 3, 5, and 8. Eleven candidate gene models were identified: *Zm00001d027619*, *Zm00001d028088*, *Zm00001d031641*, *Zm00001d031660*, *Zm00001d032024*, *Zm00001d02549*, *Zm00001d041844*, *Zm00001d053432*, *Zm00001d010230*, and *Zm00001d012665*. The SYN10137-PZA00131.14 region stands out for its agronomic traits and stress resistance, with the model Zm00001d031875 highlighted as a key candidate gene for MWS resistance [71].

To date, studies on MWS are limited compared to research on other maize diseases. In the early stages of the outbreak, the disease was primarily confined to central and South American countries, which did not attract significant attention. Although the outbreak in southern China was widespread and seriously harmful, its relatively short duration is another reason for the limited research conducted on it. Changes in the climate and the environment may not always provide the necessary conditions for its manifestation, and its occurrence is typically not annual, making it susceptible to being overlooked during cultivation. Moreover, previous studies have predominantly been conducted under natural disease conditions, with limited success in indoor isolations and inoculations. This suggests the challenging nature of artificially isolating pathogenic bacteria for this specific disease and the demanding cultivation conditions involved.

## 5. Route of Transmission and Control Strategies for MWS Disease

### 5.1. Transmission Routes

(1) Environmental medium

**Figure 4:**
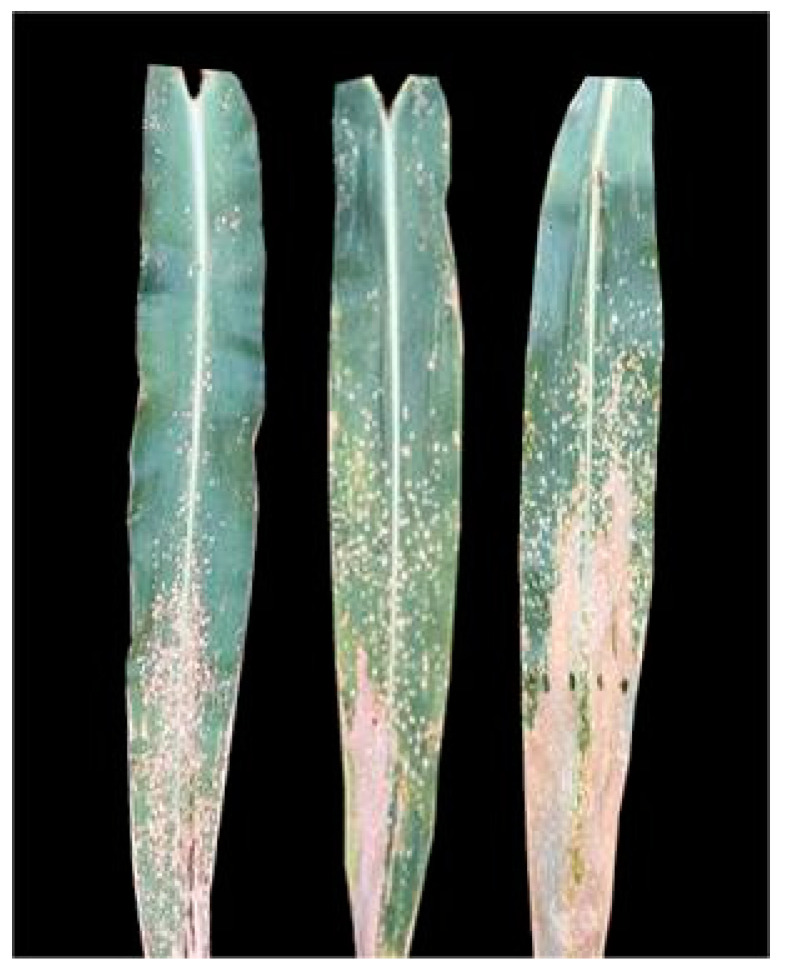
Discrepancy in disease spots on maize leaves near and far from the leaf ring end.

In selected studies conducted abroad, no significant gradient was observed between areas near the source of the inoculum and those farther away [30]. However, in the corn fields of Baoshan City, Yunnan Province, China, the disease in corn plants near the field appeared significantly and increasingly severe than in the leaves in the middle of the field [79]. Our research group also observed a similar pattern of disease distribution in the corn fields of Yanshan County, Yunnan Province. As a result, it is theorized that wind serves as the primary transmission medium for this disease in China. Initial infections often occur at the periphery of corn fields, subsequently spreading inwards. These observations differ from international reports. Additionally, in Yanshan’s corn field, a higher number of diseased spots were observed toward the end of the stretched corn leaves and far from the leaf ring (Figure 4), further supporting the idea of wind as a transmission agent. A part of these leaf blades often remains nearly horizontally oriented, and lingering water droplets may enhance the adherence of pathogenic bacteria, promoting the development of disease lesions.

Multiple studies have noted the presence of these bacteria in no-tillage soil that has been continuously planted with corn, primarily surrounding the corn’s root system [80]. These pathogens remain dormant in the soil, serving as potent sources of inoculum. Under favorable conditions, they emerge from dormancy, becoming active and dispersing MWS spores into the air. Consequently, in the following season, even non-artificially inoculated corn plants begin to exhibit the disease. It initially occurs in the leaves closest to the soil and gradually progresses upward.

(2) Plant medium

The MWS pathogen can persist in the affected regions of the plant and crop remnants, and it can also adhere to healthy corn plants [20]. The bacteria responsible for MWS can be isolated from numerous non-parasitic weeds and their seeds [20,38,80], with crabgrass being a primary bacterial carrier in Brazilian corn fields. The *Pantoea ananatis* bacteria present in these seeds show clear pathogenic effects on both oats and corn. Moreover, pathogenic endophytes have been consistently isolated from maize W22 seeds over two consecutive years [81], underscoring their potential for transmission via plant vectors.

(3) Animal transmission

*Pantoea ananatis* is prevalent within the diverse bacterial colonies inhabiting insect intestines. As many piercing-sucking insects (such as thrips and rice planthoppers) feed, they introduce these pathogenic bacteria into the plant’s phloem. This suggests the potential for these insects to act as vectors, facilitating transmission between plants [38,82].

### 5.2. Prevention and Control Strategies

Addressing the bacteria behind MWS can be intricate, given their multifaceted nature and broad infectious scope. Prior research has unveiled a plethora of germplasm resources in inbred lines that are resistant to MWS (Table 1), with considerable variation evident among descendants. This rich array paves the way for breeding resistant strains, offering not just a cost-effective and streamlined solution but also guaranteeing enduring prevention and control. Reports have shown that implementing late sowing practices in the United States effectively reduced the incidence of leaf spot disease [32], while early sowing was a key strategy for disease prevention and management in Brazil [74]. The primary objective of adjusting sowing times is to alter the presence of pathogenic bacteria in the environment and inhibit their growth under environmental pressures. The decision to change sowing times for MWS prevention and control should be based on local climatic conditions.

(1) Strengthening field management

The effective management of MWS spot disease and other leaf spot diseases requires a comprehensive approach, including deep land cultivation, appropriate crop rotation practices, and optimizing planting density.

(2) Optimizing nitrogen fertilizer application

Numerous studies have consistently demonstrated that increasing the application of nitrogen fertilizer can significantly boost maize yield. However, this increase is often accompanied by a corresponding rise in the severity of MWS. Excessive nitrogen in the environment can promote rapid plant tissue growth, the thinning of wax layers and cell walls, and reduced resistance to pathogens [83]. Therefore, the excessive use of nitrogen fertilizers should be avoided.

(3) Chemical control

The use of chemical fungicides has a significant impact on the incidence of leaf spot disease [84]. The application of the fungicide mancozeb before or during the early stages of the disease can effectively inhibit the growth of *P. ananatis* and *Phaeosphaeria maydis* [19,85]. Fungal pathogens are vulnerable to fungicidal disinfectants, whereas bacterial pathogens are sensitive to thiophanate-methyl. Treatments with disinfectants and thiophanate-methyl can effectively reduce MWS resulting from seed transmission. A study has shown that the use of oxytetracycline can effectively treat bacterial lesions and reduce fungal MWS symptoms by up to 90% [13]. Oxytetracycline possesses strong bactericidal and some fungistatic properties, making it highly effective at eliminating bacterial infections and inhibiting fungal growth. The combination of triazole compounds with cholestenes offers optimal fungicidal activity against MWS caused by *Phyllosticta maydis*, *Phoma* spp., and *Pantoea* mixed-type pathogens [84,85,86]. Zou et al. (2021) suggested that using appropriate concentrations of protective fungicides, such as azoxystrobin, ethylene imine azoxystrobin pyrazole ether fungus ester, or difenoconazole emulsifiable concentrate, during the crucial tassel emergence stage in corn cultivation can effectively reduce MWS. Additionally, adding red indole brassica can enhance efficacy and strengthen this plant’s resistance to pathogens [33].

## 6. Prospect

Identifying the pathogenic bacteria responsible for corn MWS is crucial for guiding agricultural production, given its significant impact on corn crops. Yet, the exact agents causing corn MWS globally are unclear, and there is no universally accepted naming convention. Standardizing the disease’s name, identifying the pathogenic bacteria, and discovering resistance genes for MWS may become key research areas in the future due to its pervasive nature and the evolving depth of research. Among the currently identified MWS-resistant genes, some are consistently identified, while others are closely linked to already identified genes, suggesting resistance gene clusters or pleiotropy on maize chromosomes [87]. During analyses, it is easy to miss certain QTLs with minor effects and minor genes that have critical regulatory functions [88]. This information is expected to significantly influence breeding goals and necessitate further study using advanced gene localization techniques and molecular methodologies. Plants infected by pathogenic bacteria inevitably produce toxins, but the potential risk posed by these toxins from MWS to food safety or animal health remains unclear. Our research group suggests that the transmission route of MWS in China closely resembles the pattern observed in the grassland leafy moth. It is possible that the introduction of MWS disease into China follows a similar pattern, with wind acting as the primary dispersal agent within cornfields. Further research is necessary to elucidate this disease’s transmission in maize cultivation and to devise precise prevention and mitigation strategies.

As part of the development of green agriculture, the primary approach involves the development of new maize varieties that are resistant to white spot disease. This is complemented by the implementation of other preventive and control measures, forming an integrated approach aimed at mitigating the adverse impact of MWS on maize crops. Future research should focus on understanding the occurrence pattern and etiology of MWS, conducting comprehensive analyses of the signaling and regulatory mechanisms involved in plant–pathogen interactions, and unraveling plant immune processes. Key areas for future investigation include the following: (1) collecting diverse maize germplasm resources and selecting highly MWS-resistant inbred lines for breeding disease-resistant varieties, (2) employing technologies such as GWAS, high-throughput genome sequencing, genome assembly, and transcriptome and metabolome research to map QTLs for resistance against white spot disease. This includes conducting a comprehensive effect analysis and comparative validation of these QTLs, as well as identifying potential candidate genes associated with disease resistance. (3) Utilizing methods like transposon tagging, positional cloning, and resistance gene homologous sequence (RGA) identification to expedite the cloning of disease-resistant genes, and (4) exploring the regulatory mechanisms of plant immune receptors and disease resistance genes can enhance our understanding of the plant immune system’s defense against pathogens.

In summary, we conclude that MWS is highly likely to be a bacterial maize leaf spot disease caused by *Pantoea ananatis*, although a consensus on this matter has not yet been reached. The pathogenesis and plant immune processes may share similarities with other maize leaf spot diseases. This pathogen can be transmitted through the environment, plants, and animals, making it preventable and controllable by strengthening field management and chemical measures. Plant resistance is a preferred method for controlling MWS. Significant progress has been made in identifying resistant maize inbred lines, QTL mapping, and other genetic analyses. Hopefully, with the aid of resistance breeding using new biotechnological tools and techniques, we can provide highly resistant maize lines to farmers, helping them reduce the yield losses caused by MWS in maize.

## Figures and Tables

**Table 1:** Resistant sources to corn MWS reported across different countries.

Serial Number	Country	Units	Resistant Material
1	Brazil	Home of Agriculture	CO 42, IAC Taiúba, P3041, AGM 2007, C 805, P3051, C425, Dina 70, C 701, Dina 170, XL 380 [72]
2	USA	North Carolina State University	Inbred lines with C103 or Mo17 as paternal, A619, NC258, LH213 [3,73]
3	USA	Campinas Agricultural Research Institute	L5 and L8 of CIMMYT [68]
4	Brazil	Faculty of Agronomy, Federal University of Rio Grande do Sul	LA06, LA30, AS3466, AS3477 [23,69]
5	Brazil	Syngenta Seeds LTD	DAS95, DAS41, DAS86, DAS72, DAS2 [70]
6	Brazil	Faculty of Agricultural Sciences, Federal University of Amazon	DAS95, DAS72 [66]
7	South Africa	University of KwaZulu-Natal African Centre for Crop Improvement	B23, B22, B16, CML488, CML444 [4]
8	USA	Campinas Agricultural Research Institute	PM518, IP4035, IP398 [67]
9	Brazil	Department of Agronomy, National Agricultural University of Maringa	13 hybrids (27, 22, 05, 01, 25, 26, 18, 17, 16, 20,13, 19 and 23), IAC 112, AUDPC [25]
10	Brazil	University of Sao Paulo	L08-05F [18]
11	Brazil	Federal University of Visosa	BRS1030, BRS1035, BRS1010, L2 [30,74]
12	South Africa	University of KwaZulu-Natal African Centre for Crop Improvement	A1220-4, N3-2-3-3, CML312, CML488 [6]
13	China	Yunnan Agricultural University	Tiandan 206, DS917, Fuyu 1388, Darwin 5 [13]
14	China	Institute of Dryland Food Crops, Guizhou Academy of Agricultural Sciences	QR27, QB512 [71]

**Table 2:** Mapping of QTLs associated with MWS resistance in maize.

Test Materials	Methods	QTL/QTN
158 of B73 × Mo17 F2:7 inbred lines	Composite Interval Mapping (CIM)	bin 1.06, bin 4.07, bin 7.01, bin 7.03, bin 8.07/8.08 [73]
F2 population of The L14-04B × L08-05F	Multiple Interval Mapping (MIM)	bin 1.03, bin 3.07~3.08, bin 4.08, bin 6.06–6.07, bin 8.00–8.02, bin 8.06–8.07 [18]
F2:3 population of L31.2.1.2 × L726	Multiple Interval Mapping (MIM)	qMWS1.06, qMWS2.06, qMWS2.07, qMWS3.08, qMWS4.05, qMWS4.09, qMWS4.10, qMWS8.03, qMWS8.05 [76]
183 Tropical popcorn inbred lines	Mixed Linear Models	bin 1.01, bin 1.05, bin 3.04, bin 4.02, bin 4.02, bin 5.03, bin 6.05, bin 7.02, bin 8.03 [77]
Hybrid offspring of 7 parents	Composite Interval Mapping (CIM)	qMws1.03, qMws1.04, qMws6.02, qMws8.05, qMws10.03, qMws10.06 [78]
143 inbred lines	Mixed Linear Models	SYNGENTA14387, PHM13619.5, PZE-101040783, SYN11249, PZE-101151153, SYN10891, SYN10137, PZA00131.14, SYN37674, PZE-102031753, PZE-103084298, ZM012464-0529, SYN30108, PZE-108057528, SYN4935 [71]

## Data Availability

Not applicable.
